# Behavioral and Brain Measures of Phasic Alerting Effects on Visual Attention

**DOI:** 10.3389/fnhum.2017.00176

**Published:** 2017-04-10

**Authors:** Iris Wiegand, Anders Petersen, Kathrin Finke, Claus Bundesen, Jon Lansner, Thomas Habekost

**Affiliations:** ^1^Center for Visual Cognition, Department of Psychology, University of CopenhagenCopenhagen, Denmark; ^2^Center for Lifespan Development, Max-Planck Institute for Human DevelopmentBerlin, Germany; ^3^Max-Planck UCL Centre for Computational Psychiatry and Ageing ResearchBerlin, Germany; ^4^General and Experimental Psychology, Department of Psychology, Ludwig-Maximilians-Universität (LMU) MunichMunich, Germany; ^5^Hans-Berger Department of Neurology, Jena University HospitalJena, Germany

**Keywords:** phasic alertness, visual attention, computational modeling, event-related potentials, event-related lateralizations, arousal, warning cue

## Abstract

In the present study, we investigated effects of phasic alerting on visual attention in a partial report task, in which half of the displays were preceded by an auditory warning cue. Based on the computational Theory of Visual Attention (TVA), we estimated parameters of spatial and non-spatial aspects of visual attention and measured event-related lateralizations (ERLs) over visual processing areas. We found that the TVA parameter *sensory effectiveness a*, which is thought to reflect visual processing capacity, significantly increased with phasic alerting. By contrast, the distribution of visual processing resources according to task relevance and spatial position, as quantified in parameters *top-down control α* and *spatial bias w*_index_, was not modulated by phasic alerting. On the electrophysiological level, the latencies of ERLs in response to the task displays were reduced following the warning cue. These results suggest that phasic alerting facilitates visual processing in a general, unselective manner and that this effect originates in early stages of visual information processing.

## Introduction

Visual attention is the cognitive function that enables the observer to select and process information, which is crucial for behaving effectively in the visual environment. Attention consists of multiple components supported by partly overlapping, but independent structures within a large brain network spanning visual-sensory and fronto-parietal control areas (Posner and Boies, 1971; Desimone and Duncan, 1995; Corbetta and Shulman, 2002, 2011; Fan et al., 2005). Accordingly, performance in visual tasks often depends on several components acting in concert, such as the overall available attentional processing capacity, as well as the relative distribution of these limited resources among multiple objects in the environment, which is controlled by selective attention (Bundesen, 1990; Fan et al., 2002). A distinction is to be made between *spatial* selective attention to object locations, e.g., in the left hemifield (LHF) vs. right hemifield (RHF), and *non-spatial* selective attention to task-relevant vs. -irrelevant object features, independently of the object position (Desimone and Duncan, 1995; Corbetta and Shulman, 2011). In addition, performance varies with an individual’s level of alertness, that is, the brain’s state of general readiness to respond to an upcoming stimulus (Posner and Petersen, 1990; Sturm et al., 1999). Two types of alertness can be distinguished (Sturm and Willmes, 2001): (i) tonic alertness, which is considered a sustained activation level over a longer period of time (Parasuraman et al., 1998); and (ii) phasic alertness, which refers to a short-lived activation increase elicited, e.g., by a non-informative warning cue preceding a stimulus (Fan et al., 2005). Phasic alerting has been shown to reduce reaction times (RT) in response to various stimuli (Coull et al., 2001; Thiel and Fink, 2007). This RT benefit has originally been attributed to faster preparation and/or execution of motor processes (Posner, 1978; Sturm and Willmes, 2001); although, equally likely, alerting effects may also originate in earlier sensory and attentional stages (Kusnir et al., 2011). Furthermore, whether and how aspects of selective attention, besides the general increase in processing speed, vary with phasic alertness, is not well understood (Weinbach and Henik, 2012).

The computational Theory of Visual Attention (TVA, Bundesen, 1990), in close relation to the biased competition account (Desimone and Duncan, 1995), describes visual selection and recognition as a competitive race between objects, in which probabilities for being selected and thereby encoded into the limited visual-short term memory (vSTM) depend on sensory evidence and observer-dependent biases. TVA’s neural interpretation (NTVA; Bundesen et al., 2005, 2011) assumes that the number and activation level of neurons representing an object is proportional to the rate at which the object is encoded into vSTM. TVA partitions attention into different components, which can be estimated based on an individual’s performance in simple psychophysical tasks. There is empirical evidence for the assumption that the TVA parameters are related to specific entities of the visual attention system from behavioral studies using TVA-based assessment (Matthias et al., 2010; Vangkilde et al., 2012; Sørensen et al., 2015), complemented by several neuroimaging and patient studies (e.g., Gillebert et al., 2012; Moos et al., 2012; Wiegand et al., 2014a,b; Chechlacz et al., 2015).

TVA parameter estimates of several spatial and non-spatial aspects of attention can be derived from performance in partial report tasks (Duncan et al., 1999). In these tasks, subjects have to identify briefly presented objects, defined as stimuli belonging to a pre-specified category. For example, the participant may be instructed to select by color by identifying red letters and ignoring blue ones. Targets and distractors are presented in either the same (ipsilateral) hemifield or in opposite (contralateral) hemifields. Task performance is based on accuracy only; thus, it is possible to investigate effects of phasic alerting on visual perceptual processes independent of effects on motor-related processes. Several parameters are estimated by modeling the observed probabilities of target identification: (i) Parameter *a*_i_ is a measure of the *sensory effectiveness* of object *i*, when the object is presented alone for a given exposure duration (ED) in an otherwise empty field. For multi-object displays, parameter *a* is considered to reflect the total visual processing capacity allocated to all objects, integrated over the effective ED. The parameter is independent of how attentional weights are distributed among the different objects in the visual field. The relative distribution of attentional weights, independent of the total visual processing capacity, is quantified in; (ii) parameter *spatial bias w*_index_, which is thought to reflect the distribution of weights between object in the LHF vs. RHF; and (iii) parameter *top-down control α*, which is assumed to reflect the distribution of attentional weights between task-relevant targets and task-irrelevant distracters (independent of the location of the object).

The cognitive specificity of TVA-based assessment has previously been used to disentangle interactions between alertness and different spatial and non-spatial components of visual attention. In line with the assumption that high alertness already fosters perceptual processing of incoming stimuli (Sturm and Willmes, 2001), phasic alerting induced by a visual warning cue was shown to increase the processing capacity (Matthias et al., 2010; Finke et al., 2012). Tonic alertness levels, by contrast, affected the spatial distribution of attentional weights: “Normally” alert young subjects showed a slight left-ward bias “pseudoneglect”, which shifted to a slight right-ward bias when their tonic alertness level was lowered after performing a sustained attention task (Matthias et al., 2009; McAvinue et al., 2012). In neglect patients, who suffer from a pathological rightward bias associated with persisting reduced tonic alertness (Robertson et al., 1998), phasic alerting helped to normalize the spatial distribution of attention to a more balanced weighting, besides the general increase in processing capacity (Finke et al., 2012). In the framework of NTVA, the phasic alerting effect was interpreted to reflect increased activation of neurons responding to the target letters’ features, originating in an early perceptual processing stage. The slower, intrinsic effects of tonic alertness on selective attention were proposed to arise in later processing stages, more likely influenced by top-down mechanisms including distributed areas in the attention network (Matthias et al., 2010).

By combining TVA-based assessment with event-related potentials (ERPs; Wiegand et al., 2014a,b) alerting effects on visual processing could be measured “online” (Luck, 2005). In a phasic alerting task where the cue and stimulus are presented in close succession, the cue- and stimulus-related response overlap considerably in the ERP (see Figure 2). Event-related lateralizations (ERLs) make it possible to isolate effects of alerting in the stimulus-related visual response. ERLs are computed by subtracting activity at electrodes over the hemisphere ipsilateral from activity contralateral to a laterally presented stimulus or performed motor response. A non-lateralized cue-related response will cancel out in the contra-minus-ipsilateral difference wave, and therefore not contaminate the ERL elicited by the stimulus. Making use of this methodology, studies focusing on alerting effects on motor processes in RT tasks showed that latencies of the stimulus-locked lateralized readiness potential (LRP), a motor-related lateralization marking the speed of response-selection, are shortened following a warning signal (e.g., Hackley and Valle-Inclán, 1998; Müller-Gethmann et al., 2003; Fecteau and Munoz, 2007; Hackley et al., 2007; Hackley, 2009). Similarly, visual ERLs could be used to isolate cue effects on visual processes (Luck et al., 2000; Töllner et al., 2012; Wiegand et al., 2013, 2015). Specifically, ERL latencies are assumed to reflect the timing of processing in retinotopically organized, extrastriate areas, in which the visual features of the to-be-encoded stimulus are represented (Woodman and Luck, 1999; Töllner et al., 2012); thus, ERL latencies may be sensitive to mark visual processing facilitation by phasic alerting. However, so far effects of warning signals on visual components have only been investigated on non-lateralized visual ERP components, and those have produced inconsistent results (for a review see Correa et al., 2006).

**Figure 1:**
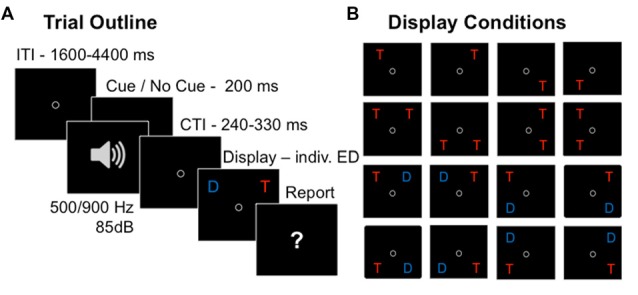
**Partial report task.** Trial sequence in the partial-report task **(A)** and 16 conditions with varying target (depicted as “T”) and distracter (depicted as “D”) configurations **(B)**.

In the present study, we analyzed accuracy scores, TVA parameter estimates, and visual ERLs in a partial report experiment, in which half of the task displays were preceded by an auditory warning cue (see Figure 1). In response to task displays with unilaterally presented stimuli, ERLs reflect sensory differences due to the overall lateralized stimulus energy in such displays, in addition to attention paid to the unilateral stimulus (Heinze et al., 1990; Mangun and Hillyard, 1990; Luck, 1995; Valle-Inclán, 1996; Hillyard et al., 1998; Hopfinger and West, 2006). Thus, ERLs in response to unilateral displays would mark cue-related modulations of both sensory and attentional effects. In displays when a lateral target stimulus is presented together with a physically similar distracter stimulus in the opposite hemifield, mere sensory differences cancel out and a cue-related modulation of the ERL in this condition (also called N2-posterior-contralateral, N2pc; Luck and Hillyard, 1994; Eimer, 1996), would solely be attributable to differences of attentional processing of the task-relevant vs. -irrelevant stimulus. Based on the previous findings, we expected that alerting would increase the total visual processing capacity, as indicated by higher parameter values of *sensory effectiveness a*. We hypothesized that the visual processing advantage would be accompanied by cue-dependent reductions of the visual ERL latencies. We also examined whether phasic alertness would affect the relative distribution of attention resources. Conceivably, if alerting changes spatial processing, reflected in parameter *spatial bias w*_index_, one might expect differences between alerting-related modulations of ERLs in response to stimuli in the LHF and RHF. If alerting affects the efficiency of task-dependent weighting, reflected in parameter *top-down control α*, one might expect that cue-related ERL modulations vary with the presence and absence of a distracter in the display.

## Materials and Methods

### Participants

Twenty-three healthy volunteers participated in the experiment. Five participants had to be excluded, one because of technical problems during the EEG recording, and four because of bad data quality and/or excessive eye-movements, leading to a rejection of more than 30% of the trials due to artifacts. In the remaining sample (*N* = 18), mean age was 25 years (SD: 2.97, Range: 20–30), six were males, and 12 females. All participants had normal or corrected-to normal vision and were not color blind. Participants did not suffer from any chronic somatic disease, or any psychiatric or neurological impairment. Before engaging in the experimental task, all participants reported to be alert, according to a subjective rating taken from the visual analog scale implemented in the CANTAB (Cambridge Cognition). The study was approved by the Health Research Ethics for the Capital Region of Denmark (De Videnskabsetiske Komiteer for Region Hovedstaden). This study was carried out in accordance with the recommendations of The Regional Committee on Health Research Ethics with written informed consent from all subjects according to the Declaration of Helsinki II, which was obtained before the experiment was carried out and the participants received gift cards (600 DKK) for their participation.

### Procedure

The PC-controlled experiment was conducted in a dimly lit, sound-proof and electrically shielded cabin. Stimuli were presented on a CRT 17″ monitor (1024 × 768 pixel screen resolution; 100 Hz refresh rate). Participants were seated in a comfortable chair at a viewing distance of approximately 90 cm from the screen. Each participant completed two experimental sessions on two separate days, conducted at the same time of day to avoid daytime influences. Each session lasted about 1.5 h. Participants were given standardized written and verbal instructions, and example displays were presented on the screen to illustrate the task before the experiment started.

On each trial (Figure 1A), either a single target, two targets, or a target and a distracter were presented. Two letters were either presented vertically (ipsilateral display) or horizontally (contralateral display), but never diagonally, resulting in 16 different display conditions (Figure 1B). A trial started with a circle presented in the center of the screen, which participants were instructed to fixate throughout the whole trial. Then the letter array was presented on a gray background, with an individually adapted ED, which was determined in a pre-test prior to the experiment (see below). In random order and in 50% of the trials, the letter array was preceded by an auditory warning cue (a 85 dB tone presented equally often with a pitch of 500 Hz and 900 Hz, varying randomly to prevent habituation effects) played for 200 ms. Participants were told not to pay attention to the warning cue while performing the partial report task. Their task was to verbally report all the red target letters (i.e., up to two in the two-target conditions, and at most one in all other conditions), and to ignore the blue distracter letters when present. The report could be performed in any (arbitrary) order and without emphasis on response speed. Participants were instructed to report only those letters they had recognized “fairly certainly” and refrain from pure guessing. The experimenter entered the responses on the keyboard and pressed a button to initiate the next trial. In order to avoid response preparation (Vangkilde et al., 2012), the inter-trial intervals (ITIs) were drawn from a geometrical distribution with a constant hazard rate of 1/3 and a range of 1600–4400 ms using time-steps of 200 ms (Figure 1A). The cue-target intervals (CTIs) were uniformly distributed with a range of 240–330 ms using time-steps of 10 ms. In trials without cue, time intervals identical to the CTIs were added to the ITIs to keep timing constant over conditions.

In each of the two sessions, a total of 800 trials were run divided into 20 Blocks, with 40 trials each. Conditions were balanced across blocks and each subject was presented with the same displays in a different random order. Letter stimuli were presented in Arial font 16, with equal frequency at each of four possible display locations forming an imaginary square, with a distance of approximately 8 cm from the fixation circle. The red target color and the blue distractor color were equiluminant (2.1 cd/cm^3^, measured with ColorCAL MKII Colorimeter (Cambridge Research Systems). The letters of a given trial were randomly chosen, without replacement, from a pre-specified set (ABDEFGHJKLMNOPRSTVXZ).

At the beginning of the first experimental session, a pre-test was conducted to practice the partial-report task and determine the ED or the test individually for each participant. First, 32 trials (two of each display condition) with a fixed ED of 40 ms were run to familiarize the participant with the trial procedure. Then a calibration procedure containing 48 trials followed, of which half were dual-target trials. Based on performance in the 24 dual-target trials, the ED was adapted stepwise: when the participant reported both targets correctly in a given trial, ED was decreased by 10 ms in the following trial; when the participant reported one letter correctly, the ED was kept at the current value; and when the participants reported no letter correct, the ED was increased by 10 ms. After this, another 48 trials were then run with the ED identified by the calibration and performance was monitored. The ED was kept when performance was 60%–90% correct in single-target displays and more than 50% correct for individual targets in dual-target displays. Else, the calibration procedure was repeated until the criterion (60%–90% correct in single-target displays and >50% correct for individual targets in dual-target displays) was reached.

Participants’ mean ED was 39.66 ms (SD: 13.76) and ranged between 20 ms and 70 ms among participants. Note that EDs were individually determined to control for potential individual differences in task difficulty due to variations in perceptual threshold; although variation in baseline performance could not be fully eliminated (Supplementary Figure S2). In any case, the EDs were short enough to prevent participants from performing micro saccades, which could contaminate the ERLs (Luck, 2005). ERLs were previously shown to be unaffected by variations in short EDs up to 200 ms (Brisson and Jolicoeur, 2007), and ERL latencies obtained in the present study were not significantly correlated with individual EDs (all *r* < 0.20, all *p* > 0.20).

### Parameter Estimation

TVA parameters were derived by modeling individual report accuracy across the different partial-report conditions (Figure 1B) by a TVA-based algorithm using a maximum likelihood method (see Dyrholm et al., 2011; Kyllingsbæk, 2006; for detailed descriptions of the algorithms). We fitted TVA parameters reflecting different aspects of spatial and non-spatial attention separately based on performance in trials with and without a warning cue. Parameter *sensory effectiveness*, *a*, is interpreted to reflect the total visual processing capacity at a given ED and is independent of how attentional resources are divided across different objects in the visual field. In more detail, *a* is the total visual processing capacity integrated over the time of the stimulus’ effective ED (see, e.g., Duncan et al., 1999; Petersen et al., 2012)^1^. The *spatial bias* parameter*, w*_index_, is considered to measure the distribution of attentional weights across the left (*w*_left_) and the right (*w*_right_) visual hemifield and is defined as the ratio *w*_left_/(*w*_left_ + *w*_right_). *w*_index_ is independent of the overall processing capacity and reflects relative weightings between objects in the LHF and RHF. A value of *w*_index_ = 0.5 indicates balanced weighting, a value of *w*_index_ > 0.5 indicates a leftward bias, and a value of *w*_index_ < 0.5 indicates a rightward spatial bias. Finally, the *top-down control* parameter, *α*, is assumed to reflect the task-related differences in weights for targets (*w*_T_) and distracters (*w*_D_), and is defined as the ratio *w*_D_/*w*_T_. Theoretically, perfect selection would imply that all attentional weight was on targets and none on distracters, resulting in *α* = 0. By contrast, unselective processing would imply equally weighted target and distracter processing, resulting in *α* = 1. Accordingly, lower *α* values indicate more efficient top-down control.

### EEG Recording

The EEG was recorded using a Biosemi amplifier system (Amsterdam, BioSemi Active 2) from 64 active Ag-Cl electrodes mounted on an elastic cap, placed according to the International 10/10 system (American Electroencephalographic Society, 1994). Five additional electrodes were placed on the left and right mastoids, at the outer canthi of the eyes (horizontal electro-oculogramm, HEOG), and beneath the left eye (vertical electro-oculogramm, VEOG). The signal was recorded at a sampling rate of 512 Hz bandwith DC-100 Hz) and referenced online to a CMS-DRL ground, which drives the average potential (i.e., common mode voltage) as close as possible to the AC reference voltage of the Analog-to-digital box (see http://biosemi.com for an explanation of the Biosemi system). For offline processing and analyses of the EEG data, we used the EEGlab (Delorme and Makeig, 2004) and ERPlab software (Lopez-Calderon and Luck, 2014). The continuous signal was filtered offline with 0.1 high-pass filter and re-referenced to the averaged mastoids. An Infomax Independent Component Analysis (Bell and Sejnowski, 1995) using the runica algorithm implemented in EEGLAB (Delorme and Makeig, 2004) was run to identify and backtransform ocular artifacts (Jung et al., 2000). The EEG was segmented into epochs of 2 s (from −1 s prior to and 1 s following stimulus display onset) for ERL analyses. Trials with signals exceeding ± 100 μV in the time window −200 ms to 800 ms on any of the electrodes were discarded as artifacts. In addition, we rejected trials in which the signal exceeded ± 50 μV on the HEOG channels.

### Analyses of Event-Related Lateralizations

**Figure 2:**
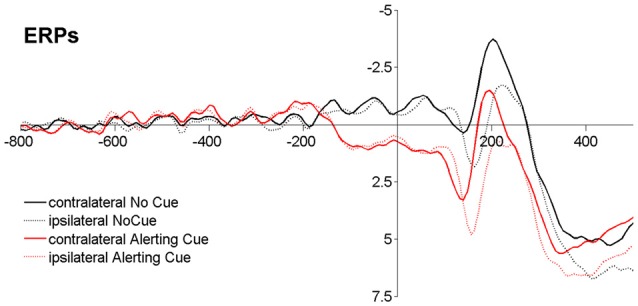
**Event-related potentials (ERPs) time-locked to stimulus onset averaged across different display conditions (Baseline −800 ms to −600 ms).** Note that, although the cue-target interval (CTI) was jittered according to common methodological recommendation for dealing with ERP overlap (Luck, 2005), the cue-related activity goes over into the stimulus-related response.

We analyzed trials with unilateral displays, in which either a single target, or a target and a distractor, or two targets were presented in the same hemifield. For these displays, ERLs indicated the contra-vs. ipsilateral differences resulting from both the sensory and attentional imbalance between the two hemifields. We also analyzed trials with bilateral displays, in which a target and a distractor were presented in opposite hemifields. For these displays, the sensory input from each hemifield is balanced and thus the contra- vs. ipsilateral differences result from purely attentional differences. In bilateral displays with two targets in opposite hemifields, no ERL could be determined as both sensory input and attention to the targets is bilaterally distributed and thus no contra-minus-ipsilateral activity could be measured reliably; these trials however were important for the TVA-based fitting of the behavioral data. Only trials in which letters were reported correctly were included in the analyses. Epochs were averaged separately for trials in which the target was on the left side and trials in which the target was on the right side in the different conditions (Figure 1B). We computed ERLs separately for LHF and RHF in four types of conditions: a single target was presented, two targets appeared in the same hemifield, a target was accompanied by a distracter in the same or opposite hemifield. ERLs were calculated by subtracting ERPs at electrodes ipsilateral from those at electrodes contralateral to the target(s), averaged over presentations in the upper and lower visual field. In the ERLs, cue-related activity in the ERPs canceled out in the contra-minus-ipsilateral subtraction procedure (Figure 2), which enabled us to investigate how alerting modulated visual perceptual and attentional processes under the given display conditions in the partial report.

No temporal smoothing (low-pass filter) was applied. We examined peak latencies of negative ERLs on pooled posterior-occipital electrodes over the left (PO7/O1) and right (PO8/O2) hemisphere. We used the ERPlab measurement tool to determine the maximum negative peak. Specifically, a peak was defined as the most negative value in the specified time window. If this value is non-zero, the procedure identifies the point that is larger than the voltages in the one sample of either side of the peak, and larger than the average of two sample points on each side of the peak. For each subject and each condition, a peak could be identified by this method. In unilateral displays, in which either a single target, a target and a distracter, or two targets were presented in the same hemifield, we determined peak latencies in the time window 120–210 ms. In bilateral displays, in which a target and a distractor were presented in opposite hemifields, peak latencies were determined in the time window 180–290 ms. We also performed analyses on ERL latencies following the jackknife procedure (Miller et al., 1998), which is reported in the Supplementary Results (Supplementary Table S1).

### Statistical Analyses

TVA parameters are considered latent parameters. That is, specific entities of the processing system operating at any instance, which are inferred from modeling the observed report accuracy. Goodness-of-fit was quantified as the squared correlation between observed performance and predicted performance by the model (*R*^2^). To test the assumption that the parameters reflect (at least partly) independent entities of the visual system, we computed Pearson correlations between the parameter estimates.

To test the effects of alerting on estimates of *sensory effectiveness a, spatial bias w*_index_, and *top-down control α*, we computed paired *t*-tests (significance 2-tailed). Report accuracy (mean scores) were entered into a 5 × 2 × 2 repeated-measure ANOVAs with the factors Display Condition (1T, 2T ipsi, 2T contra, TD ipsi, TD contra), Alerting Cue (No Cue, Cue), and Hemifield (RHF, LHF). Significant main effects revealed by the ANOVAs were followed-up by paired *t-tests* (significance 2-tailed). Before applying the parametric test, we tested that mean scores were normally distributed by Shapiro-Wilk tests (all *D*_(17)_ < 0.90, all *p* > 0.05). ERL latencies were entered into a 4 × 2 × 2 repeated-measure ANOVAs with the factors Display Condition (1T, 2T ipsi, TD ipsi, TD contra), Alerting Cue (No Cue, Cue), and Hemifield (right, left). Significant main effects and interactions revealed by the ANOVAs were followed-up by paired *t*-tests (significance 2-tailed). Finally, to evaluate the relationship between the behavioral and electrophysiological alerting effects, we computed Pearson correlations of the difference scores; that is, the relative increase of *sensory effectiveness a* by phasic alerting *a*_Cue_ − *a*_NoCue_, and the relative reduction of ERL latencies by phasic alerting (ERL_NoCue_ − ERL_Cue_) in the four display conditions.

## Results

### Report Accuracy

**Figure 3:**
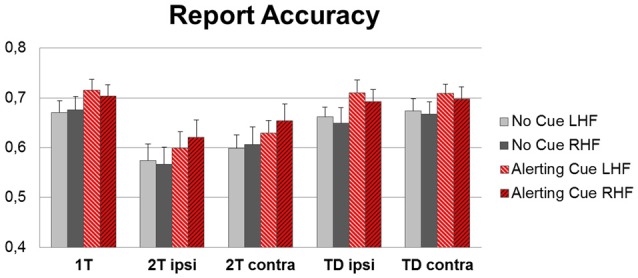
**Group mean accuracy (percentages of correctly identified target letters) for left hemifield (LHF) and right hemifield (RHF), separately for the five display conditions (1T: single-target letter, 2T ipsi: target plus second target in the ipsilateral hemifield or 2T contra: target plus second target in the contralateral hemifield, TD ipsi: target plus distractor in ipsilateral or TD contra: contralateral hemifield), for trials with (red bars) and without (gray bars) an alerting cue preceding the display**.

Phasic alerting increased the report accuracy across all conditions (Figure 3). The ANOVA on mean scores revealed a main effect of Cue (*F*_(1,17)_ = 55.68, *p* < 0.001; $\eta_{\text{p}}^{2}$ = 0.76), reflecting that more targets were correctly identified when the display was preceded by a warning cue compared to when no cue was played. There was further a main effect of Condition (*F*_(4,68)_ = 26.75, *p* < 0.001; $\eta_{\text{p}}^{2}$ = 0.61), resulting from higher report accuracy for targets presented alone (single target condition) as compared to targets presented with another target (dual target condition; both *t*s_(17)_ > 6.00, *p*s < 0.001; *d*_z_s > 1.45), and higher report accuracy for targets presented together with a distracter (target-distracter conditions) than for a targets presented together with another target (dual target condition; all *t*s_(17)_ > 3.00, all *p*s < 0.01; all *d*_z_s = 1.40). The main effect of Hemifield was not significant (*F*_(1,17)_ < 0.01, *p* = 0.88, $\eta_{\text{p}}^{2}$ = 0.02), implying similar accuracy levels for identifying targets in the LHF and RHF. No interaction was found significant (all *F* < 1.00, all *p* > 0.40).

Note that the increase in report accuracy could not be explained by simply higher report rates because of more guessing in the cue conditions (Table 1). The total number of reported letters was higher in the cued compared to the uncued trials (significantly in the target-distracter conditions (both *t*s > 2.5, both *p*s ≤ 0.05, both *d*_z_s > 0.60); marginally significantly in the single target condition and dual target condition with targets in opposite hemifields (both *t*s > 2.0, both *p*s ≤ 0.06, both *d*_z_s > 0.45); and not significantly in the dual target condition with targets in the same hemifield). However, less errors were made in the cued compared to uncued trials, in all display conditions (all *t*s > 2.5, all *p*s ≤ 0.05, all *d*_z_s > 0.50; see Table 1).

**Table 1:** **Mean number and standard deviation (in parentheses) of all reported letters (total), incorrectly reported letters (errors), and correctly reported letters (score) in the different display conditions of the partial report task**.

Condition		Total	Errors	Score
1T	*No Cue*	0.84 (0.11)	0.16 (0.08)	0.68 (0.13)
	*Cue*	0.85 (0.10)	0.14 (0.06)	0.70 (0.11)
2T ipsi	*No Cue*	0.78 (0.12)	0.17 (0.06)	0.61 (0.13)
	*Cue*	0.73 (0.15)	0.13 (0.11)	0.60 (0.11)
2T contra	*No Cue*	0.74 (0.14)	0.15 (0.06)	0.59 (0.13)
	*Cue*	0.76 (0.14)	0.12 (0.08)	0.64 (0.12)
TD ipsi	*No Cue*	0.83 (0.11)	0.17 (0.06)	0.66 (0.13)
	*Cue*	0.86 (0.08)	0.16 (0.06)	0.70 (0.11)
TD contra	*No Cue*	0.84 (0.10)	0.17 (0.06)	0.67 (0.12)
	*Cue*	0.86 (0.08)	0.16 (0.06)	0.70 (0.10)

*1T: single target letter, 2T ipsi: target plus second target in the ipsilateral hemifield, 2T contra: target plus second target in the contralateral hemifield, TD ipsi: target plus distractor in the ipsilateral hemifield, TD contra: target plus distractor in the contralateral hemifield, separately for trials with (cue) and without an alerting tone (no cue)*.

### Parameter Estimates

**Figure 4:**
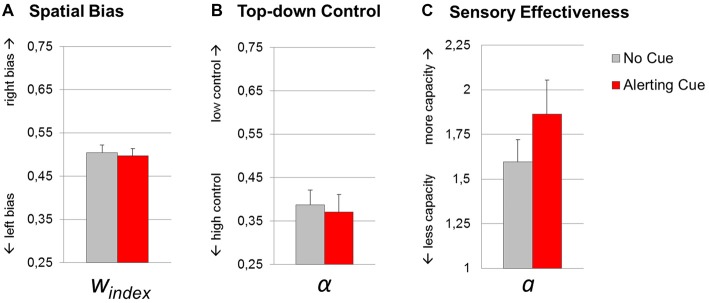
**Theory of visual attention (TVA) parameter estimates resulting from fittings of trials with (red bars) and without (gray bars) an alerting cue.** Group mean values of parameters spatial bias *w*_index_ **(A)**, top-down control α **(B)** and sensory effectiveness *a* **(C)**. Error bars indicate standard errors of the mean.

The *t*-tests revealed a significant effect of the warning cue on parameter *sensory effectiveness a* (1.60 (0.52) vs. 1.86 (0.81); *t*_(17)_ = 2.49, *p* = 0.02; *d*_z_ = 0.59). By contrast, *top-down control α* (0.39 (0.14) vs. 0.37 (0.17); *t*_(17)_ = 0.44, *p* = 0.67; *d*_z_ = 0.10), and *spatial bias w*_index_ (0.50 (0.07) vs. 0.50 (0.07); *t*_(17)_ = 0.53, *p* = 0.60; *d*_z_ = 0.13) did not significantly change with the cue manipulation. Thus, while phasic alerting increased the total visual processing capacity integrated over time, the relative distribution of attentional weights with respect to objects’ spatial location and task-relevance, were not sensitive to changes in alertness (Figure 4).

The overall model fit was good for the condition with alerting cue (*R*^2^ = 0.86) and without cue (*R*^2^ = 0.80). In accordance with the assumption that the parameters are related to specific aspects of visual processing, we did not find significant correlations between top-down control *α* and spatial bias *w*_index_ (*r* = 0.17, *p* = 0.50), top-down control *α* and sensory effectiveness *a* (*r* = 0.31, *p* = 0.20), or between spatial bias *w*_index_ and sensory effectiveness *a* (*r* = 0.15, *p* = 0.54).

### ERL Peak Latencies

**Figure 5:**
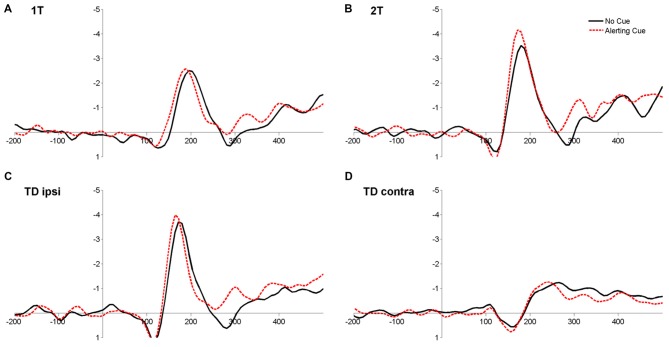
**Grand-average event-related ipsi-minus-contra lateralizations at parieto-occipital electrodes (PO/O) for the four display conditions 1T: single target letter (A)**, 2T: target plus second target in ipsilateral hemifield **(B)**, TD ipsi: target plus distractor in ipsilateral hemifield **(C)** and TD contra: target plus distractor in the contralateral hemifield **(D)** for trials with (red dashed line) and without (black solid line) an alerting cue, averaged over LHF and RHF.

The ANOVA on latencies revealed a significant main effect of Cue (*F*_(1,17)_ = 15.70, *p* = 0.001, $\eta_{\text{p}}^{2}$ = 0.48), reflecting that ERLs peaked earlier when target displays were preceded by a warning cue compared to when no cue was played (Table 2; Figure 5). In addition, the main effect of Condition (*F*_(3,51)_ = 120.40, *p* < 0.001, $\eta_{\text{p}}^{2}$ = 0.88) was significant. Latencies in the condition with bilateral target-distracter conditions, in which no lateralization due to sensory differences in hemifields occurs, were longer than in all unilateral display conditions (all *t*s_(17)_ < 9.00, all *p*s < 0.001; all *d*_z_s > 2.00; see Table 2). Latencies in the condition with a target and distracter in the same hemifield were further shorter than latencies in the single target and dual target conditions (both *t*s_(17)_ < 4.00, *p*s < 0.01; both *d*_z_s > 0.95), and latencies in the dual target condition were shorter than in the single target condition (*t*_(17)_ = 2.78, *p* = 0.013; *d*_z_ = 0.66; see Table 2). The main effect of Hemifield was not significant (*F*_(1,17)_ = 0.12, *p* = 0.73, $\eta_{\text{p}}^{2}$ = 0.02). There was further a significant interaction between Cue and Condition (*F*_(3,51)_ = 3.16, *p* < 0.05; $\eta_{\text{p}}^{2}$ = 0.16). The alerting effect on latencies was numerically present in all conditions, but was more pronounced in the target-distracter conditions (both *t*s_(17)_ > 3.00, both *p*s < 0.01; both *d*_z_s > 0.74), than in the single and dual target conditions (both *t*_(17)_ < 1.5, *p* > 0.10; both *d*_z_s < 0.30). No other interactions were significant (all *Fs* < 0.5, all *ps* > 0.6; see Figure 5).

**Table 2:** **Mean and standard error of the mean (in parentheses) of ERL latencies measured in four display conditions of the partial report task**.

Condition		ERL latencies
1T	*No Cue*	185.55 (4.50)
	*Cue*	181.64 (3.97)
2T ipsi	*No Cue*	178.17 (3.92)
	*Cue*	173.83 (2.70)
TD ipsi	*No Cue*	172.09 (2.37)
	*Cue*	154.51 (4.41)
TD contra	*No Cue*	252.17 (5.90)
	*Cue*	230.47 (6.00)

*1T: single target letter, 2T ipsi: target plus second target in the ipsilateral hemifield, TD ipsi: target plus distractor in the ipsilateral hemifield, TD contra: target plus distracter in the contralateral hemifield, separately for trials with (cue) and without an alerting tone (no cue)*.

The increase of *sensory effectiveness a* by phasic alerting (*a*_Cue_ − *a*_NoCue_) correlated significantly with the reduction of ERL latencies by phasic alerting (ERL_NoCue_ − ERL_Cue_) in the single target condition (*r* = −0.56, *p* = 0.02), however, not with cue-related ERL latency differences in the other display conditions (all *r*s < −0.20, all *p*s > 0.40; see also Supplementary Results, Figure S1).

## Discussion

The present study shows a selective effect of phasic auditory alerting on visual processing capacity, as reflected in higher values of the TVA parameter *sensory effectiveness a*. By contrast, phasic alerting did not change the spatial distribution of attention or task-related weighting, as indicated by similar parameters values of *spatial bias w*_index_ and* top-down control α* in cue and no-cue conditions. This behavioral pattern concurred with shorter latencies of ERLs in response to the task display of the partial report preceded by a warning cue compared to when no cue was played.

### Alertness Affects Early Visual Processing Stages

While previous behavioral studies have suggested that visual processing is facilitated by phasic alerting (Matthias et al., 2010; Kusnir et al., 2011), this study demonstrates that a behavioral benefit following a warning signal concurs with a reduction of early visual ERL latencies. This latency reduction was found in all display configurations of the partial report experiment, which corresponds to the behavioral finding that phasic alerting did not change the hemi-spatial and task-related relative distribution of processing resources, but rather facilitated visual sensory and attentional processing in an unselective fashion. The reduction of ERLs by phasic alerting in the single target condition was further correlated with the relative increase in the parameter *sensory effectiveness a*.

This finding is in line with a recent extension of (N)TVA that formalized how the level of alertness, *A*, affects the processing rate of categorizations in terms of TVA’s equations (Bundesen et al., 2015). Increasing alertness *A* is considered to increase the neural activation representing all visual categorizations proportionally with a common factor. This implies that any categorizations of the form “object x has feature i” are made faster when subjects are more alert. The relative distribution of attentional resources among objects, governed by observer-dependent biases to particular object features (such as color or location), by contrast, would be unaffected by changes in the general alertness level.

Of note, the correlation between the alerting effect on sensory effectiveness and ERL latencies was found significant only for the single letter condition, and the data were too weak to show statistical significance in (less powerful, but more robust) non-parametric correlational analyses (see Supplementary Results). A replication of correlation analyses should be attempted in future studies with larger samples sizes and/or even more trials, which would increase the signal-to-noise ratio in the EEG data. The effect of alerting on ERLs was further stronger in conditions where a distracter was present compared to when only targets were present (although this effect was not significant in the analyses following the jackknifing procedure, see Supplementary Results, Table S1). However, neither report accuracy in conditions with vs. without distracters were affected differently by the cue, nor was the parameter top-down control *α* (which is assumed to represent differences in the processing of targets in the presence vs. absence of distracters) modulated by alerting. Thus, other sources of variance that do not manifest in the behavioral measures, for example, varying sensory inputs in the display configurations, contributed to the variance in ERL latencies and need to be further explored.

In addition, a more fine-grained level of analysis could be achieved by distinguishing whether the processing benefit reflected in the increased *sensory effectiveness a* results from an increase in the processing rate of visual categorizations, a lowered perceptual threshold, or both (Bundesen, 1990; Petersen et al., 2017). In the present partial report experiment and other near-threshold visual tasks with one fixed ED for each participant (e.g., Kusnir et al., 2011), it is not possible to separate the individual’s visual processing rate from the threshold for conscious perception and, in case of unmasked displays, the duration of iconic memory. Consequently, only the total amount of processing, parameter *a* (i.e., the accumulated number of categorizations at this particular ED), can be measured (for details see Bundesen, 1990, 1998; Kyllingsbæk, 2006). A systematic investigation using a TVA-based paradigm with varying EDs and masked display conditions could provide a more precise distinction between phasic alertness effects on the perceptual threshold and visual processing speed.

### Phasic Alerting, Intrinsic Arousal and Temporal Preparation

While we found that only the total amount of visual capacity was affected by phasic alerting, some previous TVA-based studies found diverse effects also on the attentional parameters reflecting the relative distribution of processing resources. The different findings likely result from varieties in the task designs. In particular, temporal contingencies between the warning signal and stimuli, the CTI, seem to interact with alerting effects on selective processing components. In experiments with randomly varying CTIs, a modulation of spatial processing biases (Matthias et al., 2010; CTIs 80–650 ms) and top-down control (Ásgeirsson and Nieuwenhuis, 2016; CTIs 30–300 ms) induced by the cue were found, in addition to the total processing advantage. However, these effects disappeared in conditions with largely constant CTIs of 200–300 ms, while the general processing facilitation remained; which is in line with the results obtained in our study. A possible explanation for the interactions of temporal contingencies and effects on selective attention might be that under conditions of varying CTIs, a gradual build-up of expectancy for the imminent emergence of the stimulus alters the subject’s intrinsic arousal state (Matthias et al., 2010). We assume that this endogenous alertness state is related to voluntary temporal preparation and affects selective attentional processing (Matthias et al., 2009; Vangkilde et al., 2012, 2013; Sørensen et al., 2015). Under conditions as in the present study, where the CTI is largely constant and informs the subject to respond to a stimulus after a short, highly predictable time period, the intrinsic arousal level is likely stable and “pure” phasic alerting is observed.

It has been suggested that phasic alertness already imposes a general processing advantage at early stages in the visual processing stream (Matthias et al., 2010; Vangkilde et al., 2012). The present approach enabled us to support this assumption and isolate the early alerting effect on both the cognitive and electrophysiological level. Previous ERP studies did not consistently report such modulations on early visual potentials by warning cues (for a review see Correa et al., 2006; Nobre, 2010). Notably, in these studies, phasic alerting co-occurred with effects of voluntary temporal preparation. As mentioned above, this likely affected later attentional components influenced by top-down mechanisms implemented via distributed areas in the attention network. Furthermore, the analyses of lateralized activation to overcome the problematic overlap of cue- and stimulus-related activation has been introduced only in the present study, while this methodological problem was not addressed in those earlier studies. Future studies may now systematically investigate visual ERLs under different CTI and display conditions to dissociate voluntary temporal preparation and phasic alerting effects on total processing capacity and selective distribution of resources.

### Conclusion and Outlook

By combining TVA-based parametric assessment of attentional components with EEG, we demonstrated that phasic alerting facilitated early visual processing and increased the total amount of processing resources for object recognition. By contrast, phasic alerting did not change the relative distribution of attentional resources among stimuli with regard to their spatial position or task-relevance, indicating an unselective boost of resource deployment. In our study, participants were young, healthy and thus, presumably capable of maintaining a stable state of intrinsic alertness to optimally benefit from the warning cue. Quite possibly, in groups with abnormal or more varying intrinsic alertness levels, such as neurological patients, psychiatric patients with attention-deficit disorders, or elderly individuals, phasic alerting may also affect components of selective attention. Specifically, in patients with brain injury suffering from lowered levels of intrinsic alertness and visual hemi-neglect (Robertson et al., 1995; Husain and Rorden, 2003), phasic alerting has been shown to change the spatial weighting towards a more normal distribution of processing resources (Finke et al., 2012; see also Robertson et al., 1998). Studying individual differences in intrinsic and phasic alerting on attentional components by the present TVA-EEG approach would be useful to better understand the neuro-cognitive mechanisms underlying the complex interactions between attention and alertness.

## Author Contributions

IW, AP, CB, KF and TH contributed to the resarch idea and designed the study. AP programmed the experiment. IW and AP analyzed the data. IW and JL collected the data. IW wrote the manuscript. AP, TH and CB revised the manuscript.

## Conflict of Interest Statement

The authors declare that the research was conducted in the absence of any commercial or financial relationships that could be construed as a potential conflict of interest.

## Footnotes

^1^According to TVA, performance for a target presented alone at a location *i* is given by *P*_i_ = 1 − exp (−*a*_i_; Bundesen, 1990). Here, *a*_i_ = *s*_i_ (*t − t*_0_). For a constant exposure duration, *a* is thus proportional to *s*, and can be taken as a measure of sensory effectiveness (see Duncan et al., 1999).
