# Supplementary material for: Behavioral and Brain Measures of Phasic Alerting Effects on Visual Attention
Source: Front Hum Neurosci. 2017 Apr 10;11:176. doi: 10.3389/fnhum.2017.00176 (PMC5385325; doi:10.3389/fnhum.2017.00176)
Supplement: Supplementary file 1 [file DataSheet_1.pdf]

Supplementary Results

Behavioral and brain measures of phasic alerting effects on visual attention

Iris Wiegand, Anders Petersen, Kathrin Finke, Claus Bundesen, Jon Lansner, Thomas Habekost

Frontiers in Human Neuroscience

1. ERL analyses following the jackknifing procedure

We repeated analyses on ERL latency measures following the jackknifing procedure introduced by Miller et al., 1998 (see also Kiesel et al., 2008; Ulrich & Miller 2001), an alternative measure of the timing of ERPs, assumed to be less prone to noise-related distortions than single-participant measurements. With the jackknife approach, latencies are measured from *n* grand averages, computed from subsamples of *n*-1, with each participant being omitted from one of the subsample grand averages. We analyzed i) peak latencies and ii) fractional peak latencies (i.e., the time point where the voltage reached 80% of the maximum peak) in the time windows 120-210 for unilateral displays and 180-290 for bilateral displays.

In the analyses on peak latencies after jackknifing, the same descriptive pattern of results was found as reported in the main article (see Table below), however, neither the main effect of Cue [$F(1,17)=391.22$, $F_c(1,17)=1.35$, $p=.26$], nor the main effect of Condition [$F(1,17)=639.06$, $F_c(1,17)=2.23$, $p=.15$], nor the interaction of the two factors [$F(1,17)=63.73$, $F_c(1,17)=0.22$, $p=.64$], did survive the *F*-value correction, $F_c = F/(n\text{-}1)$. The analyses on fractional peak latencies, by contrast, revealed a significant main effect of Cue [$F(1,17)=2276.91$, $F_c(1,17)=7.88$, $p=.01$] as well as of Condition [$F(1,17)=3923.98$, $F_c(1,17)= 13.58$, $p=.002$], but no significant interaction of the two factors [$F(1,17)=291.51$, $F_c(1,17)=1.01$, $p=.32$].

The additional analyses support the critical finding of a cue-related reduction in ERL latencies which we found in the latency measures reported in the main article. They do not confirm the interaction between cue effects and conditions, suggesting that noise contributing to the variance in ERL latency measures affected the cue-related effect in the varying display conditions differently.

Table

Mean and standard errors of the mean of the ERL peak and onset latencies measured in four display conditions of the partial report task (1T: single target letter, 2T ipsi: target plus second target in the ipsilateral hemifield, TD ipsi: target plus distractor in ipsilateral, TD contra: target plus distracter in contralateral hemifield), separately for trials with (cue) and without an alerting tone (no cue) following the jackknife-procedure (Miller et al., 1998).

Condition		ERL peak latencies	ERL fractional peak latencies
1T	*No Cue*	199.22 (0.01)	176.87 (3.22)
	Cue	190.97 (0.30)	167.75 (0.92)
2T ipsi	*No Cue*	181.64 (0.57)	161.02 (1.67)
	Cue	175.13 (0.35)	156.25 (0.01)
TD ipsi	*No Cue*	172.09 (0.22)	156.25 (2.37)
	Cue	140.63 (0.01)	125.00 (0.01)
TD contra	*No Cue*	248.70 (4.50)	230.90 (7.20)
	Cue	214.64 (0.50)	191.84 (1.84)

2. Correlation analyses

We found a significant correlation (Pearson) between the cue-related increase in parameter *sensory effectiveness a* and the cue-related ERL latency reduction in the single target condition, but not with ERL latency reductions in the other conditions (see Figure 1). We further explored whether cue-effects on the other parameters *top-down control α* and *spatial bias* w_{index} may have masked the correlations between cue effects on parameter *a* and ERL latencies in the target-distracter and dual target conditions. This does not seem to be the case: Partial correlations including cue-effects on *α* and w_{index} as covariates did not change the pattern of results. The partial correlations between the effects of cue on *sensory effectiveness a* and ERL latency reduction in the single target condition was significant [r=.58, p=.02], but not in the other conditions [all rs<.30, all ps>.30].

One participant had overall larger *a*-values and also showed a stronger cue-effect on *a* (Figure 1). Non-parametric (Spearman Rho) correlation analyses between the cue-related increase in parameter *sensory effectiveness a* and the cue-related ERL latency did not approach significance in the single target condition [r=.26, p=.30], or any other condition [all rs<.15, all ps>.50]. However, importantly, the ANOVAs on parameter estimates and ERLs revealed the same pattern of results when this participant was excluded [ME Cue $F(1,16)$=1.07, p=.31; alpha: ME Cue: $F(1,16)$=0.04,

p=.85; ERLs: ME Cue $F(1,16)=17.56$, p=.001; ME Condition $F(1,16)=114.07$, p<.001, Cue x Condition: $F(1,16)=2.66$, p=.06)], implying that the effects were not driven by a single observation that is clearly different from the others in the data set.

Finally, we explored the relationship between baseline performance (report accuracy in the single target condition) and the cue effect on parameter *sensory effectiveness a*. We found a marginal significant positive correlation between the individual alerting effect on *a* and baseline accuracy [parametric (Pearson): r=.43, p=.08; non-parametric (Spearman Rho): r=.46, p=.06], reflecting that participants with lower baseline performance tended to benefit less from the cue. It would be interesting to further explore this relationship in future studies designed to systematically test the influence of baseline performance on phasic alerting. Notably, in this study individual differences in baseline performance were partly cancelled out by individually adjusting the exposure durations.

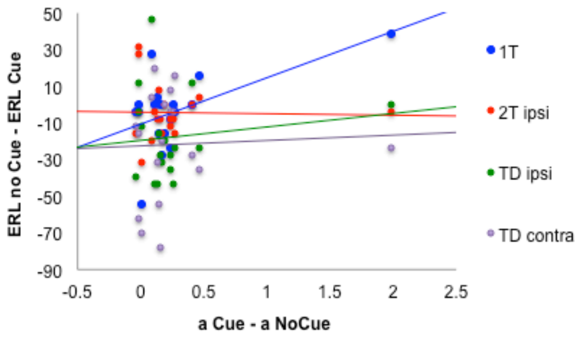

Figure 1: Correlations between the cue-related increase in sensory effectiveness ($a_{Cue} - a_{NoCue}$) and the relative reduction of ERL latencies by phasic alerting ($ERL_{NoCue} - ERL_{Cue}$) in the four display conditions (1T: single-target letter alone, 2T ipsi: target plus second target in ipsilateral hemifield, TD ipsi: target plus distractor in ipsilateral hemifield, and TD contra: target plus distractor in the contralateral hemifield.

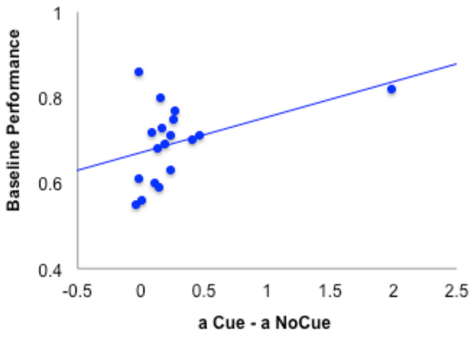

Figure 2: Correlations between the cue-related increase in sensory effectiveness ($a_{Cue} - a_{NoCue}$) and baseline performance in the single target condition.

References

Kiesel, A., Miller, J., Jolicœur, P., & Brisson, B. (2008). Measurement of ERP latency differences: A comparison of single-participant and jackknife-based scoring methods. *Psychophysiology*, *45*(2), 250-274.

Miller, J., Patterson, T., & Ulrich, R. (1998). Jackknife-based method for measuring LRP onset latency differences. *Psychophysiology*, *35*(1), 99-115.

Ulrich, R., & Miller, J. (2001). Using the jackknife-based scoring method for measuring LRP onset effects in factorial designs. *Psychophysiology*, *38*(5), 816-82.
